# Causes and Treatment of Obesity

**Published:** 1872-03

**Authors:** W. T. Taylor

**Affiliations:** Fishersville, Ky.


THE GEORGIA
MEDICAL COMPANION:
A MONTHLY ADVISER.

VOL. 2. ATLANTA, GA., MARCH, 1872. NO. 3

### PART I.

### Original Communications and Special Selections.

### CAUSES AND TREATMENT OF OBESITY.

BY W. T. TAYLOR, M. D., FISHERSVILLE, KY.

"To be or not to be" fat is a subject that seems to have been almost entirely ignored by modern medical writers, although all medical men are aware that an excess of fat is the prolific cause of an infinity of morbid conditions of the system.

Thus who is not cognizant to the fact that obesity is a common cause of sterility, both in man and beast. What medical man does not know that an excess of fat causes the human epidermis to crack, mottling the skin with white speckles and streaks; that it induces hernias of the most distressing forms; that it is the parent of ulcerated legs; that it gives rise to headaches, giddiness and dimness of sight; and more than anything else, that it predisposes to that terrible form of disease—apoplexy.

The causes of obesity are various. First, there is a natural and constitutional tendency to fat. Obesity may be hereditary. Almost every one is born with a certain predisposition, which is written on his countenance. Out of every hundred persons who die of consumption, ninety have brown hair, long faces, and sharp noses. Out of every hundred obese persons, ninety have short faces, round eyes, and obtuse or snub noses. It is a fact then, that there are individuals predestinated to obesity, whose digestive organs elaborate an extra quantity of fat.

You will sometimes notice in society, a lively little girl, with rosy cheeks, a roguish nose, plump hands, short, broadish feet, and rounded proportions generally. The prophetic medical sage, beholds her, as she will be ten years hence, and sighs over the full-blown expansion to which her form will become developed. Perhaps her mamma sits beside her, to tell you what she will be, without the exercise of second sight.

Secondary causes of corpulence are, long indulgence in sleep, in bed, and constant riding in carriages, to the exclusion of walking exercise. The Bedouin Arab who is always abroad, and active to procure the means of his nomad existence, is said never to be fat; nor is the toiling laborer, who lives on half a dollar a day, and who earns it.

Other causes of obesity are a great fondness for farinaceous, starchy and sugary diet; want of thought, as is manifested in the puffy condition of many idiots; a great absorption of fluids, whether water, beer, tea or preparations of milk,—or by frequent tepid baths, or even by constantly breathing damp air, or such as is slightly surcharged with carbonic acid, and deficient in oxygen. At every inspiration the more oxygen that is taken in, the more carbon (one of the elements of fat) is thrown off from the lungs, and consequently from the system. The inhabitant of the clear, pure atmosphere of the mountain, is rarely so fat as the resident in the moister stratum which fills the valley.

But the grand cause of obesity is our eating and drinking more than enough. It has been said, that one of the privileges of the human race is to eat without being hungry, and to drink without being dry. This double propensity is found wherever men exist. Savages indulge in it to a brutal extent, whenever they have the opportunity; and it is undeniable that we, members of civilized society, both eat and drink too much. At weddings, at political feats, at banquets, enormous quantities of eatables and drinkables are consumed, of which our bodily frames stand in no real need. Such of us as have good stomachs, convert the surplus into fat, while those who have bad ones, transmute it into indigestions, colics and sometimes even worse forms of disease.

Now, in view of the present inconvenience and frequent distress of obesity, and the probable future ills to which its possessor is exposed—is it possible to diminish, or to remove entirely *embonpoint*, without injuring the health?

An answer to this question is of practical importance in another direction. So long as obesity is undesirable and ungraceful, just so long will attempts be made by ignorant persons to get rid of the superabundant fat, by means that will certainly destroy health, and jeopardize life. How many young persons have fallen victims to the marasm brought on by daily-doses of vinegar, taken with the object of making themselves thinner. Vinegar and all other acids can produce leanness (when they do not cause death) only by deranging the general health through the injury they cause to the digestive canal. A persistence in drinking strong lemonade as an habitual beverage, has proved scarcely less injurious. As to the use of iodine and iodide of potassium to diminish fat, the practice should be condemned by physicians in the strongest terms, as being productive of the most fatal consequences.

As to the question, whether *embonpoint* can be diminished without injury to the health, we think we can give an affirmative answer.

To accomplish this, the grand principle to be considered in the treatment, is to put the patient on a regimen of *meat principally*, with the *smallest* possible amount of fluid, and that not water. Of course, we do not mean that the patient is to have *absolutely* nothing but meat; but we do mean, that meat is to be the principal article of diet, and that other things are to be used very sparingly.

In a hundred parts of human fat there are seventy-nine of carbon, fifteen and a fraction of hydrogen, and five and a fraction of oxygen. But water is nothing but the protoxide of hydrogen; and hydrogen is one of the main elements of fat. The patient, therefore, must eat but few vegetables, or watery messes, or hotrolls, puddings, tarts, potatoes, sweet-biscuit, apple-rolls, nor cakes in any of their protean forms; because, all of those dainties have carbon and oxygen for their principal bases. If he will persist in living on leguminous, farinaceous and liquid diet, he will make fat as certainly as the bee makes honey by sucking the flowers.

Chemistry proves that the principal base of meat is *azote*, which does not enter into the composition of fat; while the chief elements of fruits, sugar, flour and starch are carbon and hydrogen, the elements of fat. Human fat is found ready-made in certain aliments which are not flesh, as in olive oil, and in all the oleaginous seeds. If you live on lean meat, you will not fatten so fast as those who follow a regimen composed of carbonic and hydrogenic bases.

In conjunction with diet, other means may be brought into action which will aid in the decomposition of fat. The alkalies, combine with fat to form soap. Such alkalies administered in ordinary doses, never produce inconvenience; they increase rather than diminish the appetite, and thus favor the decrease of fat. Dr. Cullen, in his Elements of Medicine, relates that a physician named Fleming, sometimes succeeded in reducing embonpoint by prescribing soap-pills. Another English writer speaks highly of alkaline baths as an antidote to obesity; while a French practitioner records a case of emaciation resulting from the use of carbonate of soda and soda-water, which she was ordered to take with a different object in view.

It is not to be understood that alkalies alone will deliver the patient from his burden of fat. If, by diet, he takes in as many grease-making elements as the alkali drives out, things will remain in their old condition, the supply being equal to the demand. Even when living exclusively on meat, he may spoil all by drinking too much. The absorption of the smallest possible quantity of liquid, is an indispensible condition, whether in the form of food, drink, or baths. A moist atmosphere even encourages the growth of fat; some people become sensibly heavier in muggy weather.

But if the patient can be induced to follow strictly the regimen prescribed—restricting himself, for example, to a beefsteak or a couple of cutlets, with a very small quantity of vegetables, and a small cup of coffee for breakfast—the same for dinner, with the exception of the coffee, for which, milk or wine might be substituted—and for supper a cutlet, with a small cup of tea, and be careful between meals, to drink but little water, beer or other liquid, and if, with this regimen, he will take four or five grains of the bi-carb. of sodæ or carb. of potass. after each meal, he will almost as certainly diminish, in size, as will the statue of snow which has been modeled by some youthful Powers, under the genial rays of a mild winter-day's sun.

Most fat people are enormous water-drinkers, or what is nearly as bad, beer-drinkers. Let them be restricted to two or three glasses of liquid, a day. When thirsty, let them drink but a very small quantity at a time. Much relief will be experienced, whenever a wish to drink is felt between meals, by rinsing the mouth frequently with water, either pure or slightly acidulated with vinegar.

The alkali should be used which is especially indicated in the case under treatment. The dose employed, also, should be graded according to circumstances surrounding and controlling the particular case.

If the patient, be of sedentary habits, or is naturally lazy (as is *sometimes* the case) he should be directed to take as much exercise in the open air as possible, when the weather is favorable.

If the above plan of treatment, which we have so briefly, and so imperfectly sketched, be followed strictly, fat patients need not despair. Although the restrictions may be thought by them to be unnaturally rigid, yet, in a few weeks, if followed up, they will be cheered and comforted by noting a sensible diminution of their abdominal equator, and a decided mitigation of other distressing symptoms, and be made to rejoice that they can again stoop down and "unloose the latchet of their own shoes" without the intervention of a careless servant or a scolding wife.

We hope that what we have written may serve, at least, to draw out some abler pen among the contributors to THE COMPANION, as we honestly believe, that a class of patients of the *weight*, and dignity, and *solid* qualities, generally, of those to which we have called attention, should not be ignored longer, but that this morbid condition of a respectable class of our fellow-men should have that *generous* consideration which a subject of so much *magnitude* and *gravity*, would naturally call forth.

